# Development and psychometric validation of a core competency scale for military nurses in high-altitude extreme environments

**DOI:** 10.3389/fmed.2026.1791003

**Published:** 2026-04-13

**Authors:** Shijie Fang, Ruixuan Zhao, Dongwen Li, Lin Cui, Jimin Feng, Hongxu Wang, Xiaoqiong Xiong

**Affiliations:** 1 North Sichuan Medical College, Nanchong, Sichuan, China; 2 General Hospital of Western Theater Command, Chengdu, Sichuan, China; 3 Chengdu Medical College, Chengdu, Sichuan, China

**Keywords:** competency, high altitude, military nurses, onion model, reliability and validity testing, scale

## Abstract

**Background:**

With the ongoing evolution of military operations, future high-altitude missions are expected to present significant challenges due to extreme environmental and geographical conditions. As essential members of the medical support system, military nurses play a crucial role in various combat rescue operations—particularly in casualty care under special environmental conditions—where their competency directly influences the effectiveness of medical support. Evaluating casualty care capabilities in extreme settings not only reflects the overall performance of rescue personnel but also provides a foundation for capability development and training. This study developed a core competency scale for high-altitude extreme environment rescue among military nurses (HA-MNCS) and examined its reliability and validity, offering a practical tool for use in training and assessment by managers.

**Method:**

Based on a literature review and semi-structured interviews, an initial item pool was constructed. The scale was refined through expert consultation, group discussions, and a pilot survey. Using convenience sampling, 220 active-duty or civilian nurses from two hospitals in China were recruited. Item analysis, reliability, and validity tests were conducted on the collected questionnaire data.

**Results:**

The final version of the scale consists of 36 items across four dimensions: theoretical knowledge, professional skill, comprehensive ability, and personal trait. The item-content validity index (I-CVI) ranged from 0.833 to 1.000, and the scale-content validity index (S-CVI) was 0.978. Exploratory factor analysis (EFA) revealed that Bartlett’s sphericity test *χ*^2^ value was 3217.183 (*p* < 0.001), the KMO coefficient was 0.839, and the cumulative variance explained by the four common factors was 70.040%. The results of confirmatory factor analysis (CFA) indicated that the root mean square error of approximation (RMSEA) was <0.08, the root mean square residual (RMR) was <0.05, and the incremental fit index (IFI) and comparative fit index (CFI) were both >0.9. The Cronbach’s *α* coefficient of the scale was 0.948, the split-half reliability was 0.810, and the test–retest reliability was 0.962.

**Conclusion:**

The HA-MNCS demonstrates good reliability and validity and can serve as an effective tool for evaluating the core competency of military nurses in high-altitude extreme environment rescue operations. It provides a valuable reference for improving assessment systems and optimizing training programs.

## 1 Background

Against the backdrop of the ongoing evolution of the global military landscape, future high-altitude military operations may face multiple challenges posed by extreme natural environments and complex geographical conditions (1, 2). First, high-altitude regions generally exhibit harsh natural characteristics such as low oxygen levels, low temperatures, strong ultraviolet radiation, and low atmospheric pressure, which can significantly impair human respiratory, circulatory, blood, and nervous systems (3, 4). Research indicates (2, 5, 6) that low-oxygen and low-temperature environments can inhibit platelet aggregation function while promoting excessive red blood cell proliferation, leading to coagulation disorders and increased bleeding risks, posing a serious threat to the lives of injured personnel. Additionally, when personnel rapidly ascend to altitudes above 3,000 meters, they are prone to altitude sickness, high-altitude pulmonary edema (HAPE), and high-altitude cerebral edema (HACE), all of which significantly increase the complexity and urgency of medical support (7, 8). Secondly, high-altitude regions typically feature complex terrain and inadequate transportation infrastructure, compounded by year-round snow, ice, and extreme cold weather, which greatly hinder the rapid evacuation and timely transfer of injured personnel, directly impacting the timeliness of medical treatment (2). Thirdly, modern military operations exhibit characteristics of all-domain deployment and multi-terrain application, manifested in dispersed battlefield spaces, diverse injury factors, complex injury types, surging medical demands, and an increasing proportion of non-combat injuries (9–11). This multi-domain hybrid operational style imposes higher demands on the medical support system (12): on one hand, medical personnel must possess stronger comprehensive capabilities. Only well-trained, multi-skilled medical teams can swiftly and accurately assess injuries and implement effective interventions in complex environments, thereby ensuring the efficient operation of the medical rescue system; on the other hand, the medical support system must also have faster response speeds and more precise resource allocation capabilities to adapt to the demands of high-intensity, high-dynamic military operations (13, 14).

Medical support plays a crucial role in improving casualty survival rates, shortening recovery times, and maintaining sustained combat capabilities. Military nurses, as an integral part of the medical support system, play an irreplaceable role in various military tasks, including routine medical services, disaster relief, and epidemic prevention and control. Especially in the field of casualty care in extreme environments, their capabilities directly impact the quality of medical support and treatment tasks (15, 16). Military nurses must possess the ability to excel in specific environments, encompassing motivation, traits, self-awareness, values, and professional knowledge or skills. These characteristics can be quantitatively assessed through scientific methods and can significantly distinguish between high-capability and average-capability levels (17).

Research has shown (18, 19) that clear and well-defined evaluation criteria are crucial for competency-based talent development. Assessing rescue competency in extreme environments not only helps to understand the overall performance of rescue personnel, but also provides a solid foundation for their competency development and training. Several scales have been developed to assess the competencies of military nurses. The Readiness Estimate and Deplorability Index (READI), developed by Reineck et al. (20), evaluates nurses’ readiness to provide medical care in operational environments across six dimensions: clinical nursing competency, military nursing capabilities, warrior/survival capabilities, personal affairs/physical fitness/psychosocial readiness, leadership and management support, and group cohesion. While READI provides a comprehensive framework for general military deployment, it was not designed for extreme environmental conditions and lacks specific dimensions related to high-altitude physiology, hypoxia adaptation, or cold-weather injury management. Similarly, the professional competency scale for military nurses (PCSMN), developed by Chinese scholars (21), includes four dimensions: clinical nursing knowledge and skills, military nursing knowledge and skills, professional competence, and overall quality. Although this scale demonstrates good psychometric properties, its content focuses primarily on routine military healthcare settings and does not address the unique physiological and psychological challenges posed by high-altitude environments.

The structural framework of existing scales also differs significantly from the structure required in high-altitude environments. Competency in extreme conditions is not a simple aggregation of individual skills, but rather a complex interplay of personal traits, professional knowledge, and environmental stressors. This layered, multidimensional structure is accurately summarized by the “Onion Model of Competency” (22). The onion model of competency provides an appropriate theoretical framework for understanding the capabilities required of military nurses in high-altitude extreme environments. Proposed by Boyatzis, this model conceptualizes competence as a multi-layered structure: the outermost layer comprises knowledge and skills (visible and easily developed); the middle layer consists of attitudes and abilities (less observable but highly malleable); and the innermost layer represents core personality traits such as values and professional ethics (deeply ingrained and relatively stable) (23). This layered structure is particularly relevant to high-altitude military nursing contexts. The outermost layer of knowledge and skills corresponds to the foundational clinical competencies military nurses must master (24), including high-altitude pathophysiology knowledge and combat casualty care skills. These competencies can be enhanced through training or education. The intermediate layer of attitudes and competencies encompasses the cognitive and communication coordination abilities required for complex decision-making in specialized environments (25). The innermost layer of values and personal traits encompasses the psychological resilience and motivational attributes essential for sustained, effective practice in extreme conditions, such as discipline, stress resilience, mission loyalty, and the ability to maintain cognitive function under hypoxic stress (26).

Therefore, this study aims to develop and validate a context-specific tool based on the competency onion model: the high-altitude military nurse core competency scale (HA-MNCS). Unlike existing tools, the HA-MNCS specifically assesses competencies required for nurses practicing in high-altitude extreme environments. As a practical instrument, this scale can be utilized for pre-deployment competency evaluations, precise identification of training needs, and combat readiness assessments. By developing this tool, we aim to provide a scientific foundation for the training, selection, and performance evaluation of military nurses in high-altitude environments.

## 2 Method

### 2.1 Drafting of the questionnaire

#### 2.1.1 Theoretical basis

**Figure 1:**
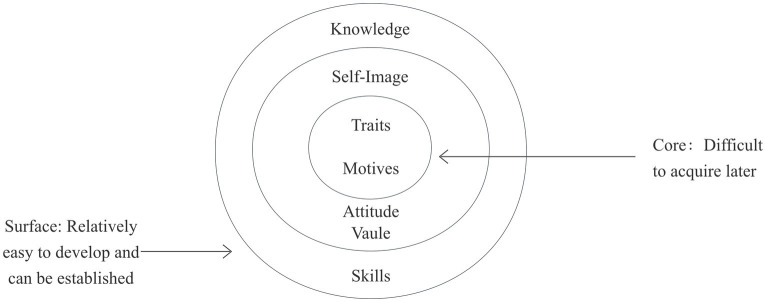
The onion model of competency.

Competency was first formally proposed by Harvard University professor David McClelland in 1973 (27). It refers to the key abilities that individuals or organizations must possess to achieve outstanding performance in a specific field, which can be measured and clearly distinguish performance levels. This study is grounded in the onion model of competency (23), which conceptualizes competency as layered from surface-level knowledge and skills to deep-seated personal traits (Figure 1). Based on this framework, we conducted semi-structured interviews with military hospital nursing staff to understand their current capabilities and needs in high-altitude extreme environment rescue, informing the development of the preliminary HA-MNCS item pool.

#### 2.1.2 Literature review

We conducted a systematic search in the Cochrane Library, PubMed, Embase, FMRS, CINAHL, PsycINFO, China National Knowledge Infrastructure (CNKI), Wanfang Database, VIP Database, and China Biomedical Database (CBM). The search period was from the establishment of the databases to May 2023. The following search terms were used in various combinations: high-altitude, qualitative research, emergency rescue, training, military nurses, civilian nurses, education, disaster, public health emergency, rescue, military, preparedness, war. Additionally, we manually searched the references of the identified studies to identify other eligible articles. Finally, the researchers conducted an in-depth reading, analysis, and interpretation of the included literature. Employing a synthesis approach, they integrated the findings to extract four competency categories: knowledge, technical skills, comprehensive abilities, and personal qualities, comprising 29 specific competency indicators (26).

#### 2.1.3 Semi-structured interviews

A purposive sampling method was employed to select doctors, nurses, and healthcare management experts with experience or theoretical knowledge in the treatment of war trauma patients from two military hospitals for semi-structured interviews. Inclusion criteria: (1) Hold a valid medical or nursing license; (2) Have participated in high-altitude medical support missions, disaster relief operations, emergency response tasks, or battlefield emergency medical training exercises; (3) Voluntarily agree to participate in this study. The interview outline was designed using the STAR tool from the Behavioral Event Interview method: (1) Have you participated in or organized any high-altitude-related missions (medical support, disaster relief, emergency response, or battlefield emergency medical training exercises)? Can you share your experiences or feelings during this process? (2) Did you have any memorable experiences during the mission? (3) What do you consider to be the greatest challenges or difficulties you faced during the mission? How did you address them? (4) During the mission, what aspects do you believe require improvement or enhancement? (5) What competencies (core competencies, core qualities) do you think military nurses should possess when providing on-site medical care in the special environment of the plateau? (6) How do you think the competencies (core competencies, core qualities) of military nurses in providing on-site medical care in the special environment of the plateau should be improved? The interview outline is available in Supplementary File 1. After transcribing the interview recordings into written materials, content analysis was employed to examine the textual data, extracting and compiling 4 primary indicators, 18 secondary indicators, and 36 tertiary indicators. Based on the theoretical framework, and integrating findings from literature reviews and semi-structured interviews, group discussions resulted in the formulation of 4 primary indicators, 21 secondary indicators, and 85 tertiary indicators.

#### 2.1.4 Expert consultation

##### 2.1.4.1 Select experts

Purposeful sampling was used to select experts for consultation. Expert inclusion criteria: (1) Bachelor’s degree or higher; (2) Intermediate-level professional title or higher; (3) At least 7 years of work experience in nursing management, military nursing, military medicine, high-altitude medicine, critical care nursing, disaster nursing, medical support, and battlefield trauma care; (4) Informed consent and voluntary participation.

A total of 16 experts participated in two rounds of expert consultation. The average age of the consulted experts was 40 ± 4.09 years, with an average work experience of 16.13 ± 5.44 years. Nine experts held associate senior or higher professional titles, and 11 held master’s degrees or higher. Their fields of expertise included military medicine (4), nursing management (4), military nursing (4), and medical support services (4). For details, see Supplementary File 2.

##### 2.1.4.2 Compilation of questionnaires and item screening methods

The questionnaire consists of three sections. The first section is the questionnaire on core competency indicators for military nurses in high-altitude extreme environments. This questionnaire uses a 5-point Likert scale to rate the importance of each indicator and corresponding item, with the following scores: very important = 5, relatively important = 4, generally important = 3, relatively unimportant = 2, and very unimportant = 1. The second round of written inquiry forms can be found in Supplementary File 2. The second part covers the experts’ basic information, including age, workplace, years of experience, education level, position, and professional title. The third part addresses the experts’ judgment criteria and familiarity with the indicators, including their familiarity with various indicators and the questionnaire on judgment criteria. Based on the expert survey results, items with importance scores ≤4.0 and coefficient of variation ≥0.25 were removed, and the content of the items was revised.

##### 2.1.4.3 Expert consultation results

This study conducted two rounds of expert correspondence surveys. In the first round, 17 expert questionnaires were distributed, with 16 returned, yielding an effective response rate of 94.12%. In the second round, 16 questionnaires were distributed, with all 16 returned, achieving a 100% effective response rate. The valid response rate for both rounds exceeded 70%, indicating a high level of expert engagement (28). The expert panel remained consistent across both rounds. After assigning values and performing calculations, the expert authority coefficient was determined to be 0.872, exceeding 0.7, suggesting a reliable level of expert authority (29). In the first round of expert correspondence, Kendall’s *W* was 0.197 with a CV range of 0.00–0.26 points. In the second round, this coefficient increased to 0.213 with a CV range of 0.00–0.16 points. Both *p*-values were <0.001, indicating progressively greater consistency in expert opinions across the two rounds (30). Based on expert feedback, the indicators underwent revisions, deletions, additions, and consolidations through group discussions. The final indicator system comprises 4 primary indicators, 21 secondary indicators, and 86 tertiary indicators.

#### 2.1.5 Group discussion

Purposeful sampling was employed to invite seven military nurses and nursing managers from two military hospitals in Sichuan Province to participate in a focus group discussion. Among them, three held bachelor’s degrees, three held master’s degrees, and one held a doctoral degree; two held intermediate-level titles, three held associate senior-level titles, and two held senior-level titles; their years of work experience ranged from 17 to 35 years. Experts discussed the wording, rationality, and feasibility of each indicator item, adjusted and refined the content of the items, and ultimately developed the HA-MNCS (Supplementary File 3).

### 2.2 Preliminary survey

A convenience sampling method was used to select 20 military nurses from a military hospital in Chengdu in May 2025 for a preliminary survey to assess the clarity and understandability of the questionnaire items. Based on pre-survey findings, appropriate adjustments and revisions were made to the questionnaire. The questionnaire achieved a 100% response rate (20/20). The questionnaire took 5–15 min to complete, with an average completion time of 12.29 min. The pre-survey revealed that two items required rewording to enhance clarity. Participants raised no major objections to the questionnaire content, which was found to be semantically clear and comprehensible (see Supplementary File 4 for the pre-survey questionnaire).

### 2.3 Validity and reliability testing

#### 2.3.1 Study population

Convenience sampling was used to select military nurses from two hospitals in Sichuan Province and Gansu Province as study participants between May and June 2025. Inclusion criteria: (1) Active-duty or civilian nurses in military hospitals; (3) Possession of the corresponding nursing practice certification; (3) Informed consent. Exclusion criteria: (1) Nurses undergoing further education, residency training, or internships; (2) Those who were absent or withdrew during the survey period. According to Kendall’s rough estimation method (31), the sample size should be 5–10 times the number of items. In exploratory factor analysis, the appropriate sample size was calculated to be 5 times the number of items (32). For confirmatory factor analysis, the sample size should exceed 200 (33). Assuming a non-response rate of 10%, a total of 220 military nurses were recruited for this study. Among them, 30 nurses completed the same questionnaire within 2 weeks.

#### 2.3.2 Survey tools

(1) General information survey form: Based on a review of the literature, a general information survey form was developed, including age, gender, educational attainment, years of work experience, professional title, position, and department. (2) Draft of the HA-MNCS: The initial scale comprises 4 dimensions and a total of 37 items (2 items were removed for content validity). It employs a Likert 5-point scoring system. For items 1 to 21, scores of 1 to 5 represent “Not mastered,” “Poorly mastered,” “Basically mastered,” “Mostly mastered,” and “Completely mastered,” respectively. For items 22 to 37, scores of 1 to 5 represent “Disagree,” “Somewhat disagree,” “Neutral,” “Agree,” and “Completely agree,” respectively. Higher scores indicate higher high-altitude medical support capabilities among military nurses.

#### 2.3.3 Project analysis

(1) Critical value method: Sort the questionnaire scores from highest to lowest, designate the top 27% as the high-score group and the bottom 27% as the low-score group, and use an independent samples *t*-test for comparison. Retain items with *t*-values >3.00 and *p* < 0.05 (34, 35). (2) Correlation coefficient method: Calculate the Pearson correlation coefficients between each item score and the total questionnaire score. Retain items with an absolute correlation coefficient >0.30 and *p* < 0.05 (36). (3) Internal consistency coefficient method: If the total Cronbach’s *α* coefficient of the questionnaire significantly increases after deleting a particular item, then delete that item (37).

#### 2.3.4 Validity testing

##### 2.3.4.1 Content validity

Six experts were invited to rate the relevance of each scale item to its corresponding dimension. The experts evaluated each item using a 1–4 rating scale, with the following options: “4 = highly relevant,” “3 = relevant but requires minor revisions,” “2 = requires major revisions,” and “1 = not relevant.” If necessary, the experts were also asked to provide explanations. Then, the values of the I-CVI and the S-CVI were determined by calculating the ratio of the number of experts who rated each item as 3 or 4 to the total number of experts. Items with an I-CVI greater than 0.78 were retained, while the rest were deleted (38).

##### 2.3.4.2 Structural validity

(1) Exploratory factor analysis (EFA): Common factors were extracted using principal component analysis and maximum variance method. Common factor extraction was based on cumulative variance contribution >50%, eigenvalue >1, and compliance with the scree plot test. When screening items, those with loadings <0.5 were removed. If dual loadings existed (both loadings >0.5 and the difference <0.15), the items were removed. If the difference >0.15 and the item had a higher loading in the original dimension, it was considered to belong to the original factor (31). (2) Confirmatory factor analysis (CFA): Chi-square/degrees of freedom ratio (CMIN/DF) <3.00; normed fit index (NFI), incremental fit index (IFI), comparative fit index (CFI), and Tucker–Lewis index (TLI) >0.90; root mean square error of approximation (RMSEA) <0.08, and root mean square residual (RMR) <0.05, the model fit meets the ideal criteria (31).

#### 2.3.5 Reliability analysis

(1) Internal consistency reliability: A Cronbach’s *α* coefficient >0.70 is acceptable, and a Cronbach’s *α* coefficient of 0.80–0.90 indicates good reliability. (2) Split-half reliability: A split-half reliability coefficient >0.80 indicates good reliability. (3) Test–retest reliability: From the 220 participants who completed the initial survey, a convenience sample of 30 nurses was selected to complete the same questionnaire again after a two-week interval. Participants were eligible for the retest if they agreed to be re-contacted and were accessible for follow-up. The retest questionnaires were distributed face-to-face using the same paper-based format as the initial survey. Participants were asked to complete the retest questionnaire within three days of the two-week mark. A test–retest reliability coefficient >0.70 indicates good stability of the scale (39).

### 2.4 Statistical analysis

Statistical analysis was performed using SPSS 27.0 and AMOS 24.0. Count data were described using counts and percentages. Continuous data that followed a normal distribution were expressed as mean ± standard deviation ( $\bar{X}\pm S$ ), while those that did not follow a normal distribution were described using median (M) and interquartile range (P_25_, P_75_). Survey data from the initial questionnaire were used for item analysis and reliability and validity testing. Differences were considered statistically significant at *p* < 0.05 or *p* < 0.01.

### 2.5 Ethics

This study was approved by the Ethics Committee of the General Hospital of Western Theater Command (Ethics Number: 2024EC4-ky014) and was conducted in accordance with the ethical standards of the Declaration of Helsinki. Written informed consent was obtained from all participants prior to their inclusion in the study.

## 3 Results

### 3.1 General information

A total of 250 questionnaires were distributed, and 220 valid questionnaires were returned, with a valid questionnaire return rate of 88.0%. Detailed information on participant characteristics is shown in Table 1. The average age of the 220 military nurses was (37.28 ± 6.88) years. Identity attributes: 34 were active-duty military nurses, and 186 were civilian nurses. Years of experience: 34 (15.5%) had ≤5 years, 58 (26.4%) had 6–10 years, 42 (19.1%) had 11–15 years, 38 (17.3%) had 16–20 years, and 48 (21.8%) had >20 years. A total of 120 participants (54.5%) reported involvement in major rescue experiences, while 100 (45.5%) had no such experience. To examine potential differences between active-duty and civilian nurses, independent-sample *t*-tests were conducted comparing their scores on each dimension of the HA-MNCS and the total scale. No statistically significant differences were found between the two groups on any dimension or total score (all *p* > 0.05). To assess the practical significance of intergroup differences, effect sizes (Cohen’s *d*) were calculated. All effect sizes were small or negligible: the effect size for the total score was *d* = 0.064; for the theoretical knowledge dimension, *d* = 0.106; for the professional skill dimension, *d* = 0.069; for the comprehensive ability dimension, *d* = 0.216; and for the personal trait dimension, *d* = 0.158 (see Table 2 for details). These findings indicate that active-duty nurses and civilian nurses demonstrate highly comparable levels of core competency in high-altitude military nursing.

**Table 1:** Demographic details of the study participants (*n* = 220).

Variables	Category	Frequency/Statistical value	Percentage
Gender	Male	27	12.3%
	Female	193	87.7%
Identity attributes	Active-duty nurses	34	15.5%
	Civilian nurses	186	84.5%
Level of education	College degree or below	20	9.1%
	Bachelor’s degree	183	83.2%
	Master’s degree or above	17	7.7%
Department	Internal medicine	108	49.1%
	Surgical	32	14.5%
	Others	80	36.4%
Marital status	Unmarried	33	15.0%
	Married	180	81.8%
	Divorced	7	3.2%
Years of experience (years)	≤5	34	15.5%
	6–10	58	26.4%
	11–15	42	19.1%
	16–20	38	17.3%
	>20	48	21.8%
Position	Nurse	119	54.1%
	Nursing manager	91	41.4%
	Others	10	4.5%
Professional title	Nurse	23	10.5%
	Senior nurse	62	28.2%
	Nurse in charge	102	46.4%
	Associate director of nurses	30	13.6%
	Director of nurses	3	1.4%
Prior major rescue experience	Yes	120	54.5%
	No	100	45.5%
Age (years)	M (P_25_, P_75_)	38 (32, 42)	—

**Table 2:** Subgroup analysis of HA-MNCS scores by identity attributes.

Dimension	Active-duty nurses ( $\bar{X}\pm S$ )	Civilian nurses ( $\bar{X}\pm S$ )	*t*	*p*	Cohen’s *d*
Theoretical knowledge	36.32 ± 5.71	35.64 ± 6.58	0.568	0.571	0.106
Professional skill	49.76 ± 8.30	49.26 ± 7.21	0.368	0.713	0.069
Comprehensive ability	30.85 ± 3.40	31.57 ± 3.30	−1.160	0.247	−0.216
Personal trait	42.71 ± 2.84	42.15 ± 3.61	0.849	0.397	0.158
Total score	159.65 ± 16.49	158.62 ± 15.92	0.345	0.731	0.064

### 3.2 Project analysis results

(1) The results of the critical ratio method analysis showed that there were statistically significant differences in the scores of each item between the high-score group and the low-score group, with t-values ranging from 4.025 to 14.043, all *p* < 0.05. (2) The results of the correlation coefficient method analysis showed that the correlation coefficients between each item and the total score ranged from 0.349 to 0.795 (*p* < 0.05). (3) The results of the internal consistency coefficient method analysis showed that the overall Cronbach’s *α* coefficient for the questionnaire draft was 0.95, and removing any single item did not significantly alter the overall Cronbach’s *α* coefficient.

### 3.3 Validity analysis results

#### 3.3.1 Content validity

The draft scale was submitted to six experts for review to assess its content validity. The I-CVI and S-CVI values were determined by evaluating the scores of all experts. As a result, two items with CVI values below 0.78 were removed from the scale. Thus, the draft scale was shortened to include 37 items. The final S-CVI value was 0.98. The I-CVI values ranged from 0.83 to 1.00.

#### 3.3.2 Structural validity

**Figure 2:**
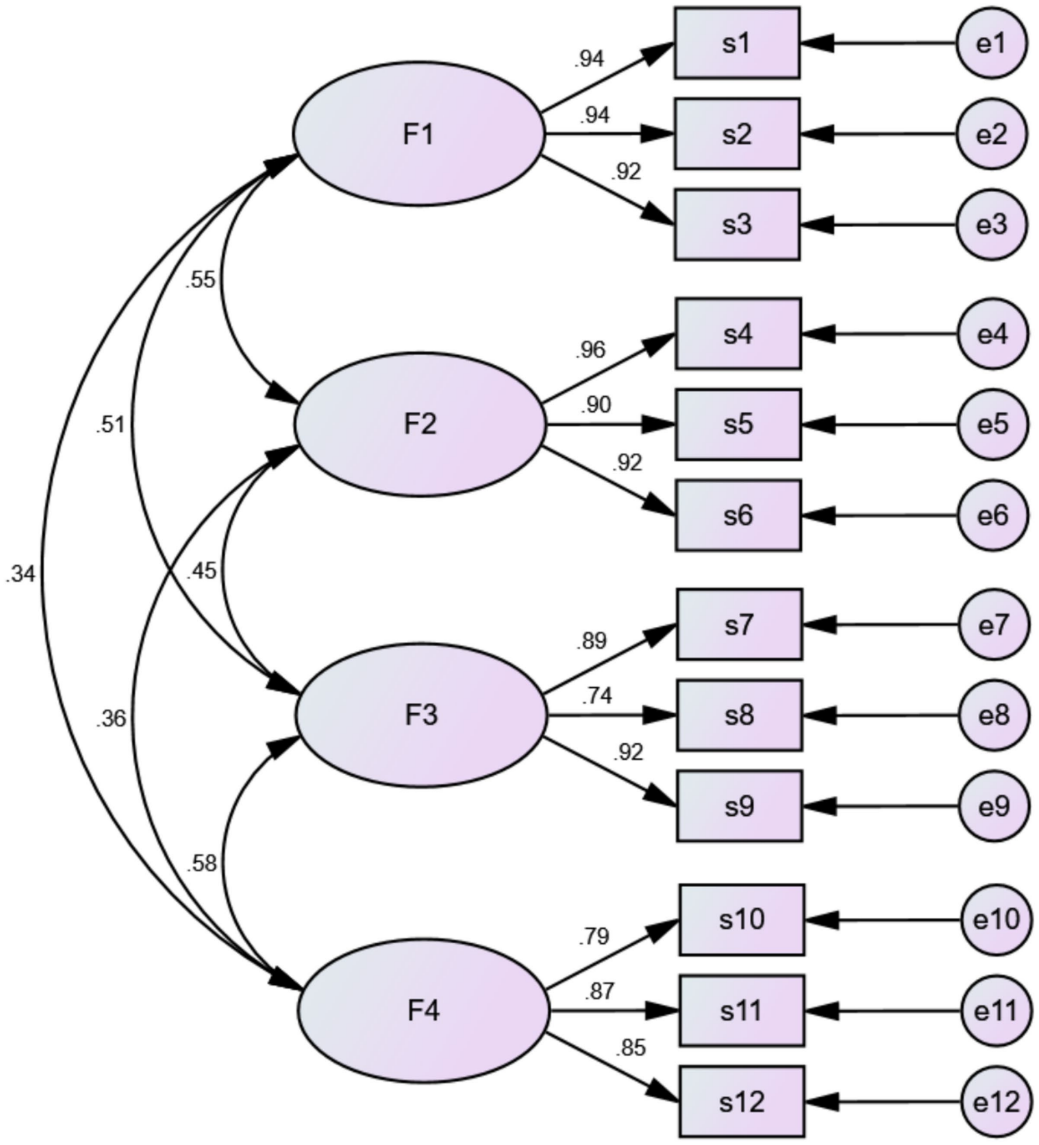
Standardized four-factor structural equation model.

(1) Using SPSS, 40% of the total sample (*n* = 88) was randomly selected for exploratory factor analysis. The Bartlett test yielded a value of 3381.741 (*p* < 0.001), and the KMO test coefficient was 0.833, >0.8 (suitable for factor analysis). Principal component analysis was performed with varimax rotation, yielding six common factors with eigenvalues >1, accounting for 77.119% of the cumulative variance. Based on the results from the competency onion model and the scatter plot, we restricted the extraction to four common factors for a second exploratory factor analysis. Item 37, “Possessing the ability to perform mental work in a high-altitude hypoxic environment,” had a cross-factor loading greater than 0.4, so it was deleted. The remaining items had loading values greater than 0.5 under their respective factors, and there were no cases of double or multiple loadings. The remaining 36 items were subjected to another exploratory factor analysis, yielding a KMO value of 0.839 and a Bartlett’s sphericity test *χ*^2^ value of 3217.183 (*p* < 0.001), indicating suitability for factor analysis. The analysis results showed that four common factors with eigenvalues >1 were extracted, with variance contribution rates of 20.397, 18.342, 15.710, and 15.591%, respectively, and a cumulative variance contribution rate of 70.040%. Based on the content of the items in each common factor, they were named the theoretical knowledge dimension, professional skill dimension, comprehensive ability dimension, and personal trait dimension, respectively. The loadings of each item on the corresponding factors are shown in Supplementary File 5. (2) The remaining 70% of the sample (*n* = 132) was used for confirmatory factor analysis to validate the four-factor structure identified in the EFA. In the initial item-level CFA, the model fit indices did not reach the commonly accepted thresholds (CMIN/DF = 2.450, IFI = 0.759, CFI = 0.756, TLI = 0.739, RMSEA = 0.105 and RMR = 0.053). This suboptimal fit is not uncommon in new scale development, as item-level data typically contain substantial unique variance and random error that can obscure model fit even when the underlying factor structure is correct (40). Importantly, all items demonstrated significant loadings on their respective factors, with standardized loadings ranging from 0.371 to 0.904 and critical ratios ranging from 4.254 to 12.786 (all *p* < 0.001), supporting the hypothesized four-factor structure (31). Given that the primary research interest was to examine the relationships among the four latent dimensions rather than to explore item-level properties, and because the dimensionality of the items had been established *a priori* through EFA and supported by significant factor loadings in the initial CFA, we employed item parceling following the recommendations of Little et al. (41) and Wu and Wen (42). Item parceling is appropriate for structural model analysis when the goal is to obtain stable and parsimonious estimates of construct-level relationships, as it reduces item-specific variance and random error (43). To ensure the suitability of this approach, we randomly grouped items within each dimension into new indicators and conducted exploratory factor analysis on the resulting parcels; factor loadings ranged from 0.726 to 0.828, confirming that the parceling was appropriate for this dataset (21, 43). After item parceling, confirmatory factor analysis was conducted on all new indicators, yielding the following results: CMIN/DF = 1.818, NFI = 0.939, IFI = 0.972, CFI = 0.971, TLI = 0.961, RMSEA = 0.079 and RMR = 0.020. The RMSEA value of 0.079 is at the upper boundary of the acceptable range, while the remaining fit indices all exceeded the recommended thresholds, indicating that the overall model fit was acceptable. Detailed results are presented in Table 3. In the four-factor model of HA-MNCS, the standardized regression weights of all items ranged from 0.74 to 0.96, indicating that each item adequately represents the corresponding factor (Figure 2).

**Table 3:** Confirmatory factor analysis of the HA-MNCS.

Fit index	Absolute fit index			Relative fit index			
	RMSEA	RMR	CMIN/DF	NFI	IFI	CFI	TLI
Judgment criteria	<0.08	<0.05	<3.00	>0.900	>0.900	>0.900	>0.900
Actual results	0.079	0.020	1.818	0.939	0.972	0.971	0.961

RMSEA, root mean square error of approximation; RMR, root mean square residual; CMIN/DF, chi-square/degrees of freedom; NFI, normed fit index; IFI, incremental fit index; CFI, comparative fit index; TLI, Tucker–Lewis index.

#### 3.3.3 Convergent validity

The average variance extracted (AVE) ranged from 0.706 to 0.866, and the construct reliability (CR) ranged from 0.878 to 0.951, indicating that the convergent validity is acceptable (44).

#### 3.3.4 Discriminant validity

The square root of the AVE is greater than the correlation coefficient between any two dimensions, indicating that the distinctive validity is acceptable (15, 44, 45), the detailed results are shown in Table 4.

**Table 4:** Correlation matrix and discriminant validity of the dimensions.

Dimension	Theoretical knowledge	Professional skill	Comprehensive ability	Personal trait
Theoretical knowledge	**0.930**			
Professional skill	0.544^**^	**0.926**		
Comprehensive ability	0.513^**^	0.448^**^	**0.855**	
Personal trait	0.340^**^	0.359^**^	0.579^**^	**0.840**

Bolded values on the diagonal are the square roots of the AVE; values below the diagonal are the Pearson correlation coefficients between dimensions; ^**^indicates *p* < 0.01.

### 3.4 Reliability analysis results

#### 3.4.1 Scale reliability

The overall Cronbach’s *α* value for the scale is 0.948, and the Cronbach’s *α* values for each dimension range from 0.896 to 0.944, all exceeding 0.8, indicating that the overall reliability of the scale and the reliability of each dimension are satisfactory.

#### 3.4.2 Split-half reliability

Split-half reliability was measured using the odd-even split-half method based on the scale items. The Spearman–Brown coefficient was 0.810, with split-half reliability for each dimension ranging from 0.795 to 0.912, all exceeding 0.7, indicating good split-half reliability and high internal consistency of the scale.

#### 3.4.3 Test–retest reliability

The recommended time interval for test–retest reliability is 1–4 weeks. In this study, the questionnaire was retested after 2 weeks for 30 survey participants. The test–retest reliability of the total scale was 0.962, and the test–retest reliability of the four dimensions ranged from 0.876 to 0.954, indicating that the scale has high consistency and stability over time, as shown in Table 5.

**Table 5:** Reliability test of the HA-MNCS.

Dimension	Cronbach’s *α* coefficient (*n* = 220)	Split-half reliability (*n* = 220)	Test–retest reliability (*n* = 30)
Theoretical knowledge	0.944	0.912	0.950
Professional skill	0.933	0.795	0.946
Comprehensive ability	0.910	0.889	0.954
Personal trait	0.896	0.806	0.876
Total score	0.948	0.810	0.962

## 4 Discussion

This study is based on the “Onion Competency Model” as its theoretical foundation. Through literature reviews, semi-structured interviews, two rounds of expert consultations, group discussions, and a preliminary survey, a draft questionnaire was developed. The process was rigorous and standardized to ensure that the questionnaire items possess high representativeness, scientific validity, and applicability. In the item analysis, all items had CR values >3.0 (*p* < 0.001), with total item correlations ranging from *r* = 0.356 to 0.795. The internal consistency coefficient analysis showed that removing any single item did not significantly alter the overall Cronbach’s *α* coefficient, indicating no need for deletion. This confirms the excellent discriminative power and homogeneity of the items. Content validity and construct validity are the most common validity evaluation indicators used to assess the validity or accuracy of a scale. Literature indicates (38) that content validity typically requires an I-CVI ≥0.78 and an S-CVI ≥0.90. In this study, the I-CVI ranged from 0.83 to 1.00, and the S-CVI was 0.98, indicating that the scale possesses high content validity. Regarding construct validity, EFA and CFA methods were employed. EFA is a commonly used research method that identifies latent common factors by analyzing the correlations among multiple observed variables, with these factors explaining the common variance of the observed variables. In this study, EFA extracted four common factors, with a KMO value of 0.839 (>0.8), a Bartlett test of 3217.183 (*p* < 0.001), and a cumulative variance contribution rate of 70.04% (>60%). The factor loadings for the remaining 36 items were all >0.6, indicating that the scale has good construct validity. In the confirmatory factor analysis of this study, RSEMA <0.08, RMR <0.05, CMIN/DF <3.00, and IFI, TLI, and CFI were all >0.900, indicating that the internal structure of the scale is acceptable. The Cronbach’s *α* for each dimension of the scale in this study ranged from 0.896 to 0.944, the split-half reliability ranged from 0.795 to 0.912, and the test–retest reliability ranged from 0.876 to 0.954, all of which were >0.7, indicating that the scale has good internal consistency and internal correlation. The results of the study indicate that the HA-MNCS has high reliability and validity.

HA-MNCS employs a competency-based onion model structured in three layers from outer to inner, decomposing core competencies into four dimensions: theoretical knowledge, professional skills, comprehensive abilities, and personal traits—totaling 36 items. The theoretical knowledge dimension, as the outermost layer of the competency onion model, consists of nine items and serves as the cognitive foundation and decision-making basis for emergency care in high-altitude extreme environments. This dimension focuses on the systematic knowledge system that military nurses must master in high-altitude special environments, covering three core areas: basic nursing knowledge, high-altitude medical nursing knowledge and high-altitude environmental characteristics, and high-altitude combat casualty treatment strategies. The professional skill dimension serves as the skill execution layer of the competency onion model, comprising 12 items. It focuses on the ability to reconstruct and adapt nursing procedures in high-altitude extreme environments, with the core objective of transforming theoretical knowledge into stable and reliable military medical rescue techniques. This dimension directly addresses the multifaceted challenges posed by high-altitude environments to conventional nursing procedures. The comprehensive ability dimension serves as the central hub for regulating competencies within the competency onion model, comprising seven components. It focuses on the dynamic integration of non-routine decision-making, resource coordination, and risk control in high-altitude military environments. The personal trait dimension serves as the core inner layer of the competency onion model, comprising eight items that encapsulate the deep-seated motivations and stable psychological traits driving military nurses to sustain rescue missions in high-altitude extreme environments. This dimension emphasizes the critical roles of environmental adaptability and psychological resilience.

The HA-MNCS differs from existing military nursing competency scales. Existing military nursing competency scales include the READI scale (20), which focuses on assessing nurses’ readiness in conventional military operational environments and primarily serves pre-deployment evaluations. The PCSMN (2022) employs a content-driven four-dimensional classification system whose structure reflects recognition of nursing competency’s multidimensionality, making it suitable for comprehensive evaluations of nursing personnel in general military medical settings (21). The HA-MNCS developed in this study deconstructs competency into three tiers: theoretical knowledge (outer layer) and professional skills (outer layer) constitute observable and trainable surface-level competencies; integrated capabilities (middle layer) involve complex decision-making and coordination management; while personal traits (inner layer) encompass deep-seated characteristics such as values, psychological resilience, and motivation. This structure reflects the developmental progression of competency from surface to core and reveals the critical role of deep-seated traits in supporting surface competencies under extreme conditions. Research indicates that at altitudes above 3,000 meters, routine nursing procedures take 1–2 times longer, cognitive function significantly declines (46, 47), and existing scales do not assess adaptability to special environments like high altitudes. Therefore, the HA-MNCS incorporates multiple plateau-specific indicators in its items, including plateau environmental characteristics and their impact on medical rescue, plateau medical support nursing knowledge, nursing knowledge for common plateau illnesses, and psychological resilience and stress coping abilities in plateau environments. These items directly address the unique challenges to nursing practice posed by plateau hypoxia, low temperatures, and complex terrain. Regarding applicability, READI and PCSMN primarily serve conventional military medical settings, whereas HA-MNCS is specifically tailored for the extreme high-altitude environment. It can be utilized for pre-deployment competency assessments, targeted training needs identification, and combat readiness evaluations. This scenario specificity positions HA-MNCS as a vital complement to existing tools.

Although the HA-MNCS was developed and validated based on a sample of Chinese military nurses, its applicability to military nurses from different cultural or organizational backgrounds warrants consideration. The challenges posed by high-altitude hypoxia, low temperatures, and complex terrain to nursing practice are universal. Therefore, the core competencies required of military nurses deployed in similarly complex special environments may share commonalities. However, caution is advised when directly applying this scale to other cultural or organizational contexts. On one hand, items involving specific military protocols, equipment, or organizational structures may require adaptation to local practices. On the other hand, differing cultural interpretations of personal traits—such as discipline and resilience—could influence item comprehension. Therefore, future research should conduct cross-national and cross-cultural validation through back-translation, expert review, and multi-group measurement equivalence testing to determine the applicability and dissemination boundaries of the HA-MNCS among international military nursing populations.

The scale developed in this study comprehensively covers the competency elements required for military nurses engaged in rescue operations in high-altitude extreme environments, representing a distinct set of indicators specific to such environments that differentiate them from military nursing work in other settings. When selecting and appointing nurses, while focusing on superficial and intermediate-level comprehensive abilities such as theoretical knowledge and professional technical proficiency, it is also important to emphasize core-level intangible traits (such as personal characteristics and motivation) to evaluate military nurses’ rescue capabilities in high-altitude environments more comprehensively and thoroughly, thereby identifying nurses with core motivation and traits.

## 5 Strengths and limitations

The core strength of this study lies in its theoretical foundation based on the competency onion model, which was developed through a systematic literature review, qualitative interviews, Delphi expert surveys, group discussions, pilot tests, and formal surveys. The scale breaks away from the traditional linear competency structure, adopting a layered approach from the inside out: “personal traits → comprehensive competencies → professional skills → theoretical knowledge.” It integrates elements such as political literacy and military obedience, demonstrating a systematic approach. In terms of empirical validation, the scale demonstrates excellent performance through rigorous reliability and validity testing. EFA identifies a four-dimensional structure, and CFA achieves acceptable model fit, with CMIN/DF values, RMSEA values, RMR values, NFI values, IFI values, TLI values, and CFI values all meeting criteria. Convergent validity, discriminant validity, total scale Cronbach’s *α* values, split-half reliability, and test–retest reliability all meet standards.

This study has certain limitations. First, convenience sampling from only two military hospitals in China restricted sample representativeness and may have introduced selection bias. Second, data collection relied on self-reporting, which could be influenced by social desirability bias, thereby affecting response accuracy. Third, although the sample size was adequate for psychometric analysis, the relatively small number of active-duty nurses (*n* = 34) limited subgroup comparisons. Future research should employ more diverse sampling strategies, including multicenter and cross-regional studies, to enhance the representativeness and external validity of the HA-MNCS. Furthermore, integrating objective performance metrics with longitudinal designs would mitigate self-report bias and assess the scale’s predictive validity for actual task outcomes.

## 6 Conclusion

This study strictly adhered to scale development guidelines to construct the HA-MNCS. Throughout the development process, rigorous quality control measures were implemented at every stage, resulting in a scale with excellent reliability and validity. It can serve as an effective tool for evaluating the emergency care capabilities of military nurses in high-altitude environments. This scale helps identify key issues in military nurses’ job performance and provides a basis for implementing competency-based training strategies, thereby promoting the development of high-level, highly adaptable nursing teams. Future recommendations include conducting further application studies of this scale in different regions to validate its cross-regional applicability and provide references for its global promotion and use.

## Data Availability

The original contributions presented in the study are included in the article/Supplementary material, further inquiries can be directed to the corresponding authors.
