# Supplementary material for: Development and psychometric validation of a core competency scale for military nurses in high-altitude extreme environments
Source: Front Med (Lausanne). 2026 Apr 13;13:1791003. doi: 10.3389/fmed.2026.1791003 (PMC13110977; doi:10.3389/fmed.2026.1791003)
Supplement: Supplementary file 1 [file Table_1.docx]

Interview Outline:

(1) Have you participated in or organized any high-altitude-related missions (medical support, disaster relief, emergency response, or battlefield emergency medical training exercises)? Can you share your experiences or feelings during this process?

(2) Did you have any memorable experiences during the mission?

(3) What do you consider to be the greatest challenges or difficulties you faced during the mission? How did you address them?

(4) During the mission, what aspects do you believe require improvement or enhancement?

(5) What competencies (core competencies, core qualities) do you think military nurses should possess when providing on-site medical care in the special environment of the plateau?

(6) How do you think the competencies (core competencies, core qualities) of military nurses in providing on-site medical care in the special environment of the plateau should be improved?
