# Supplementary material for: Development and psychometric validation of a core competency scale for military nurses in high-altitude extreme environments
Source: Front Med (Lausanne). 2026 Apr 13;13:1791003. doi: 10.3389/fmed.2026.1791003 (PMC13110977; doi:10.3389/fmed.2026.1791003)
Supplement: Supplementary file 2 [file Table_2.docx]

**Delphi Expert Consultation Questionnaire on Core Competency Indicators for High-Altitude Extreme Environment Rescue Among Military Hospital Nursing Personnel**

Instructions for Filling Out the Questionnaire:​​

This Delphi consultation questionnaire includes inquiry forms for first-level, second-level, and third-level indicators. Please judge the importance of each item according to the following scale and check (√ ) the corresponding box (select only one answer per item): “5 = Extremely Important​​, 4 = Relatively Important​​, 3 = Moderately Important​​, 2 = Relatively Unimportant​​,1 = Not Important at All​​”. If you find any item unclear, inaccurate, or believe it should be deleted, or if you think an item is categorized under an incorrect dimension, please provide your revisions in the "​​Modification Suggestions​​" column. If you believe certain content should be added or merged, please provide your suggestions in the "​​Proposed Additional Content​​" column. Please note:​​ If you recommend deleting or modifying an indicator, you are still required to provide an importance rating for that indicator. Please ensure all items are rated; do not leave any items blank or skip them.

​​Core Competency for High-Altitude Extreme Environment Rescue Among Military Hospital Nursing Personnel: First-Level Indicators Delphi Consultation Form​​

| Level 1 Indicator | Importance Score（5→1） | | | | | Note | |
| --- | --- | --- | --- | --- | --- | --- | --- |
|  |  |  |  |  |  | Delete | Modification Suggestions |
|  | Extremely Important 5 | Relatively Important 4 | Moderately Important 3 | Relatively Unimportant 2 | Not Important at All  1 |  |  |
| 1.Theoretical knowledge |  |  |  |  |  |  |  |
|  | Proposed Additional Content： | | | | | | |
| 2.Professional skill |  |  |  |  |  |  |  |
|  | Proposed Additional Content： | | | | | | |
| 3.Comprehensive ability |  |  |  |  |  |  |  |
|  | Proposed Additional Content： | | | | | | |
| 4. Personal trait |  |  |  |  |  |  |  |
|  | Proposed Additional Content： | | | | | | |

**​**Core Competency for High-Altitude Extreme Environment Rescue Among Military Hospital Nursing Personnel: Second-Level Indicators Delphi Consultation Form​​

| Level 1 Indicator | Level 2 Indicator | Importance Score（5→1） | | | | | Importance Score（5→1） | |
| --- | --- | --- | --- | --- | --- | --- | --- | --- |
|  |  |  |  |  |  |  | Delete | Modification Suggestions |
|  |  | Extremely Important 5 | Relatively Important 4 | Moderately Important 3 | Relatively Unimportant 2 | Not Important at All  1 |  |  |
| 1.Theoretical knowledge | 1.1 Military Theory and Combat Medical Support Knowledge Related to High Altitude Environments |  |  |  |  |  |  |  |
|  | 1.2 Prevention, Treatment, and Nursing Knowledge Related to Altitude-Related Illnesses |  |  |  |  |  |  |  |
|  | 1.3 Theoretical Knowledge of Field First Aid in High-Altitude Warfare |  |  |  |  |  |  |  |
|  | 1.4 Psychological Theory Knowledge |  |  |  |  |  |  |  |
|  | Proposed Additional Content： | | | | | | | |
| 2.Professional skill | 2.1 Assessment |  |  |  |  |  |  |  |
|  | 2.2 Hemostasis |  |  |  |  |  |  |  |
|  | 2.3 Airway Management |  |  |  |  |  |  |  |
|  | 2.4 Bandaging |  |  |  |  |  |  |  |
|  | 2.5 Cardiopulmonary Resuscitation (CPR) |  |  |  |  |  |  |  |
|  | 2.6 Immobilization |  |  |  |  |  |  |  |
|  | 2.7 Anti-Shock |  |  |  |  |  |  |  |
|  | 2.8 Transportation |  |  |  |  |  |  |  |
|  | 2.9 Temperature Management |  |  |  |  |  |  |  |
|  | 2.10 NBC Protection |  |  |  |  |  |  |  |
|  | 2.11 Medical Evacuation |  |  |  |  |  |  |  |
|  | Proposed Additional Content： | | | | | | | |
| 3.Comprehensive ability | 3.1 Management Coordination Skills |  |  |  |  |  |  |  |
|  | 3.2 Critical Thinking Skills |  |  |  |  |  |  |  |
|  | 3.3 Safety Management Skills |  |  |  |  |  |  |  |
|  | Proposed Additional Content： | | | | | | | |
| 4. Personal trait | 4.1 Physical Fitness |  |  |  |  |  |  |  |
|  | 4.2 Mental Fitness |  |  |  |  |  |  |  |
|  | 4.3 Military Fitness |  |  |  |  |  |  |  |
|  | Proposed Additional Content： | | | | | | | |

Core Competency for High-Altitude Extreme Environment Rescue Among Military Hospital Nursing Personnel: Third-Level Indicators Delphi Consultation Form​​

| Level 1 Indicator | Level 2 Indicator | Level 3 Indicator | Importance Score（5→1） | | | | | Importance Score（5→1） | |
| --- | --- | --- | --- | --- | --- | --- | --- | --- | --- |
|  |  |  |  |  |  |  |  | Delete | Modification Suggestions |
|  |  |  | Extremely Important 5 | Relatively Important 4 | Moderately Important 3 | Relatively Unimportant 2 | Not Important at All  1 |  |  |
| 1.Theoretical knowledge | 1.1 Military Theory and Combat Medical Support Knowledge Related to High Altitude Environments | 1.1.1 Characteristics of the High-Altitude Environment and Its Impact on the Human Body |  |  |  |  |  |  |  |
|  |  | 1.1.2 Impact of the High-Altitude Environment on Combat Wound Conditions |  |  |  |  |  |  |  |
|  |  | 1.1.3 Impact of the High-Altitude Environment on Medical Rescue Operations |  |  |  |  |  |  |  |
|  |  | 1.1.4 Knowledge Related to Health and Epidemic Prevention in High-Altitude Regions |  |  |  |  |  |  |  |
|  |  | 1.1.5 Knowledge of High-Altitude Combat Service |  |  |  |  |  |  |  |
|  |  | Proposed Additional Content： | | | | | | | |
|  | 1.2 Prevention, Treatment, and Nursing Knowledge Related to Altitude-Related Illnesses | 1.2.1 Acute Mountain Sickness (AMS) Prevention, Treatment and Nursing Knowledge |  |  |  |  |  |  |  |
|  |  | 1.2.2 High-Altitude Heart Disease (HAHD) Prevention, Treatment and Nursing Knowledge |  |  |  |  |  |  |  |
|  |  | 1.2.3 High-Altitude Polycythemia (HAPC) Prevention, Treatment and Nursing Knowledge |  |  |  |  |  |  |  |
|  |  | Proposed Additional Content： | | | | | | | |
|  | 1.3 Theoretical Knowledge of Field First Aid in High-Altitude Warfare | 1.3.1 Tactical Combat Casualty Care (TCCC) Theory |  |  |  |  |  |  |  |
|  |  | 1.3.2 Prolonged Casualty Care (PCC) Theory |  |  |  |  |  |  |  |
|  |  | 1.3.3 Preparation and Administration of Common Emergency Medications |  |  |  |  |  |  |  |
|  |  | 1.3.4 Operation and Maintenance of Common Medical Devices and Equipment |  |  |  |  |  |  |  |
|  |  | 1.3.5 Common Types of Combat Injuries and Their Clinical Manifestations |  |  |  |  |  |  |  |
|  |  | 1.3.6 Medical Response Knowledge for Injuries Caused by Specific Weapons |  |  |  |  |  |  |  |
|  |  | 1.3.7 Basic Knowledge of Nuclear Radiation Protection |  |  |  |  |  |  |  |
|  |  | Proposed Additional Content： | | | | | | | |
|  | 1.4 Psychological Theory Knowledge | 1.4.1 Theories Related to Psychological Stress Assessment |  |  |  |  |  |  |  |
|  |  | 1.4.2 Theories Related to Psychological Intervention |  |  |  |  |  |  |  |
|  |  | 1.4.3 Theories Related to Post-Traumatic Stress Disorder (PTSD) |  |  |  |  |  |  |  |
|  |  | 1.4.4 Knowledge on Psychological Impact of High-Altitude Environments and Protective Measures |  |  |  |  |  |  |  |
|  |  | Proposed Additional Content： | | | | | | | |
| 2.Professional skill | 2.1 Assessment | 2.1.1 Battlefield Environmental Assessment |  |  |  |  |  |  |  |
|  |  | 2.1.2 Rapid Casualty Assessment and Examination |  |  |  |  |  |  |  |
|  |  | 2.1.3 Triage Methodology (START) |  |  |  |  |  |  |  |
|  |  | 2.1.4 Hypoxia Status Evaluation |  |  |  |  |  |  |  |
|  |  | Proposed Additional Content： | | | | | | | |
|  | 2.2 Hemostasis | 2.2.1 Assessment of Blood Loss |  |  |  |  |  |  |  |
|  |  | 2.2.2 Identification and Management of Major Bleeding |  |  |  |  |  |  |  |
|  |  | 2.2.3 Tourniquet Hemostasis (Clamp-Type, Rotating-Type, Standard-Type) |  |  |  |  |  |  |  |
|  |  | 2.2.4 Pressure Hemostasis |  |  |  |  |  |  |  |
|  |  | 2.2.5 Wound Packing |  |  |  |  |  |  |  |
|  |  | 2.2.6 Pharmacological Hemostasis |  |  |  |  |  |  |  |
|  |  | 2.2.7 Improvised Hemostasis Methods |  |  |  |  |  |  |  |
|  |  | Proposed Additional Content： | | | | | | | |
|  | 2.3 Airway Management | 2.3.1 Airway Assessment and Clearance |  |  |  |  |  |  |  |
|  |  | 2.3.2 Manual Airway Management Techniques |  |  |  |  |  |  |  |
|  |  | 2.3.3 Use of a Bag-Valve-Mask Resuscitator |  |  |  |  |  |  |  |
|  |  | 2.3.4 Mouth-to-Mouth Resuscitation |  |  |  |  |  |  |  |
|  |  | 2.3.5 Recovery Position/Forward-Leaning Position |  |  |  |  |  |  |  |
|  |  | 2.3.6 Use of oropharyngeal and nasal airways |  |  |  |  |  |  |  |
|  |  | 2.3.7 Cricothyroid membrane puncture technique |  |  |  |  |  |  |  |
|  |  | 2.3.8 Tracheal intubation and cricothyroidotomy techniques |  |  |  |  |  |  |  |
|  |  | 2.3.9 Pneumothorax identification and management (closed, open, tension pneumothorax) |  |  |  |  |  |  |  |
|  |  | 2.3.10 Oxygen therapy techniques |  |  |  |  |  |  |  |
|  |  | Proposed Additional Content： | | | | | | | |
|  | 2.4 Cardiopulmonary Resuscitation (CPR) | 2.4.1 Timing of CPR Assessment |  |  |  |  |  |  |  |
|  |  | 2.4.2 Chest Compressions |  |  |  |  |  |  |  |
|  |  | 2.4.3 Manual or Automated Defibrillation |  |  |  |  |  |  |  |
|  |  | 2.4.4 Assessment and Advanced Life Support |  |  |  |  |  |  |  |
|  |  | Proposed Additional Content： | | | | | | | |
|  | 2.5 Bandaging | 2.5.1 Common Bandaging Methods (Triangular Bandages, Bandages, etc.) |  |  |  |  |  |  |  |
|  |  | 2.5.2 Bandaging Techniques for Common Body Areas (Head, Chest, Limbs, etc.) |  |  |  |  |  |  |  |
|  |  | 2.5.3 Bandaging Techniques for Specialized Areas (Eye, Amputations, etc.) |  |  |  |  |  |  |  |
|  |  | Proposed Additional Content： | | | | | | | |
|  | 2.6 Immobilization | 2.6.1 Cervical Collar Immobilization |  |  |  |  |  |  |  |
|  |  | 2.6.2 Limb Fracture Immobilization |  |  |  |  |  |  |  |
|  |  | 2.6.3 Spinal Fracture Immobilization |  |  |  |  |  |  |  |
|  |  | 2.6.4 Pelvic Fracture Immobilization |  |  |  |  |  |  |  |
|  |  | Proposed Additional Content： | | | | | | | |
|  | 2.7 Anti-Shock | 2.7.1 Shock Diagnosis and Assessment |  |  |  |  |  |  |  |
|  |  | 2.7.2 Determination and Evaluation of Effective Shock Resuscitation |  |  |  |  |  |  |  |
|  |  | 2.7.3 Peripheral Venous Catheterization |  |  |  |  |  |  |  |
|  |  | 2.7.4 Central Venous Catheterization |  |  |  |  |  |  |  |
|  |  | 2.7.5 Bone Marrow Canal Catheterization |  |  |  |  |  |  |  |
|  |  | 2.7.6 Fluid Resuscitation Strategies and Selection of Resuscitation Fluids |  |  |  |  |  |  |  |
|  |  | 2.7.7 Indications and Contraindications for Infusion and Transfusion |  |  |  |  |  |  |  |
|  |  | 2.7.8 Intravenous Infusion Techniques in Unstable Vibrating Dark-Field Environments |  |  |  |  |  |  |  |
|  |  | Proposed Additional Content： | | | | | | | |
|  | 2.8 Transportation | 2.8.1 Manual Transport Method |  |  |  |  |  |  |  |
|  |  | 2.8.2 Stretcher Transport Method |  |  |  |  |  |  |  |
|  |  | 2.8.3 Special Injury Transport Methods (Spinal, Cranial, etc.) |  |  |  |  |  |  |  |
|  |  | Proposed Additional Content： | | | | | | | |
|  | 2.9 Temperature Management | 2.9.1 Identification and Prevention of Hypothermia |  |  |  |  |  |  |  |
|  |  | 2.9.2 Thermal Protection |  |  |  |  |  |  |  |
|  |  | 2.9.3 Passive Warming |  |  |  |  |  |  |  |
|  |  | 2.9.4 Active Warming |  |  |  |  |  |  |  |
|  |  | Proposed Additional Content： | | | | | | | |
|  | 2.10 NBC Protection | 2.10.1 Nuclear, Chemical, and Biological Weapons First Aid Techniques |  |  |  |  |  |  |  |
|  |  | 2.10.2 Protection Against Nuclear, Chemical, and Biological Weapons |  |  |  |  |  |  |  |
|  |  | 2.10.3 Decontamination of Nuclear, Chemical, and Biological Weapons |  |  |  |  |  |  |  |
|  |  | 2.10.4 Biological Warfare Agent Decontamination and Casualty Isolation |  |  |  |  |  |  |  |
|  |  | Proposed Additional Content： | | | | | | | |
|  | 2.11 Medical Evacuation | 2.11.1 Different Evacuation Methods |  |  |  |  |  |  |  |
|  |  | 2.11.2 Indications and Contraindications for Different Evacuation Methods |  |  |  |  |  |  |  |
|  |  | 2.11.3 Monitoring and Emergency Management During Different Evacuation Methods |  |  |  |  |  |  |  |
|  |  | 2.11.4 Operation of Life Support Equipment During Medical Evacuation |  |  |  |  |  |  |  |
|  |  | 2.11.5 Air/Ground Evacuation Documentation |  |  |  |  |  |  |  |
|  |  | Proposed Additional Content： | | | | | | |  |
| 3.Comprehensive ability | 3.1 Management Coordination Skills | 3.1.1 Teamwork and Collaboration Skills |  |  |  |  |  |  |  |
|  |  | 3.1.2 Mass Casualty Management Capabilities |  |  |  |  |  |  |  |
|  |  | 3.1.3 Effective Communication Skills with Casualties and Team Members |  |  |  |  |  |  |  |
|  |  | Proposed Additional Content： | | | | | | | |
|  | 3.2 Critical Thinking Skills | 3.2.1 Ability to adjust rescue plans in real time based on battlefield conditions |  |  |  |  |  |  |  |
|  |  | 3.2.2 Ability to adapt and respond flexibly to sudden and complex issues |  |  |  |  |  |  |  |
|  |  | Proposed Additional Content： | | | | | | | |
|  | 3.3 Safety Management Skills | 3.3.1 Ability to anticipate and identify enemy fire risks |  |  |  |  |  |  |  |
|  |  | 3.3.2 Ability to anticipate and identify potential hazardous environments |  |  |  |  |  |  |  |
|  |  | 3.3.3 Professional safety self-protection capability |  |  |  |  |  |  |  |
|  |  | Proposed Additional Content： | | | | | | | |
| 4. Personal trait | 4.1 Physical Fitness | 4.1.1 Meeting Military Physical Fitness Test Standards |  |  |  |  |  |  |  |
|  |  | 4.1.2 Survival Adaptability |  |  |  |  |  |  |  |
|  |  | 4.1.3 Mental Work Capacity in High-Altitude Hypoxic Environments |  |  |  |  |  |  |  |
|  |  | Proposed Additional Content： | | | | | | | |
|  | 4.2 Mental Fitness | 4.2.1 Self-Psychological Adjustment Ability |  |  |  |  |  |  |  |
|  |  | 4.2.2 Coping Ability and Stress Resistance |  |  |  |  |  |  |  |
|  |  | Proposed Additional Content： | | | | | | | |
|  | 4.3 Military Fitness | 4.3.1 National Defense Awareness and Patriotic Consciousness |  |  |  |  |  |  |  |
|  |  | 4.3.2 Sense of Responsibility and Mission |  |  |  |  |  |  |  |
|  |  | 4.3.3 Military Knowledge and Military Proficiency |  |  |  |  |  |  |  |
|  |  | Proposed Additional Content： | | | | | | |  |

**General Information on Experts Consulted（*n*=16）**

| Variables | Category | Frequency | Percentage (%) |
| --- | --- | --- | --- |
| Age, years | $\leq$35 | 4 | 25.00 |
|  | 36～44 | 10 | 62.25 |
|  | $\geq$45 | 2 | 12.50 |
| Level of Education | Bachelor's Degree | 5 | 31.25 |
|  | Master's Degree | 5 | 31.25 |
|  | Doctoral Degree | 6 | 37.50 |
| Professional Title | Nurse in charge | 7 | 43.75 |
|  | Associate director of nurses | 8 | 50.00 |
|  | Director of nurses | 1 | 6.25 |
| Years of Experience, years | 7～10 | 4 | 25.00 |
|  | 11～20 | 10 | 62.50 |
|  | $\geq$21 | 2 | 12.50 |
| Field of Expertise | Military medicine | 4 | 25.00 |
|  | Nursing management | 4 | 25.00 |
|  | Military nursing | 4 | 25.00 |
|  | Medical support services | 4 | 25.00 |
