# Supplementary material for: Development and psychometric validation of a core competency scale for military nurses in high-altitude extreme environments
Source: Front Med (Lausanne). 2026 Apr 13;13:1791003. doi: 10.3389/fmed.2026.1791003 (PMC13110977; doi:10.3389/fmed.2026.1791003)
Supplement: Supplementary file 3 [file Table_3.docx]

The item selection and refinement process during the expert panel discussion was guided by established theories in combat casualty care. Primarily, the principles of Tactical Combat Casualty Care (TCCC) and its MARCH algorithm (Massive hemorrhage, Airway, Respiration, Circulation, Hypothermia) served as the foundational framework for evaluating the criticality of technical skills. Furthermore, the unique challenges posed by the high-altitude environment were considered through the lens of Operational and High-Altitude Medicine, ensuring the relevance of knowledge items to the specific research context.

1. Expert Panel Composition

To ensure the scientific rigor and authority of the discussion, we have assembled a panel of seven experts. Their basic information is as follows:

| Variables | Category | Frequency | Percentage |
| --- | --- | --- | --- |
| Gender | Male | 1 | 14.3% |
|  | Female | 6 | 85.7% |
| Level of Education | Doctoral degree | 1 | 14.3% |
|  | Master's degree | 3 | 42.9% |
|  | Bachelor's degree | 3 | 42.9% |
| Years of Experience (years) | 15~20 | 2 | 28.6% |
|  | 20~30 | 4 | 57.1% |
|  | ＞30 | 1 | 14.3% |
| Professional Title | Nurse in charge | 2 | 28.6% |
|  | Associate director of nurses | 3 | 42.9% |
|  | Director of nurses | 2 | 28.6% |
| Fields of Work (multiple selections allowed) | Military Care and Nursing | 7 | 100.0% |
|  | Nursing Management | 7 | 100.0% |
|  | Scientific Research | 4 | 57.1% |
|  | Clinical Nursing | 4 | 57.1% |
|  | Nursing Education | 4 | 57.1% |

2. Decision Criteria: Experts conducted evaluations based on the following four criteria.

**Relevance:** Is the item essential for measuring the target construct?

**Clarity:** Is the wording unambiguous and easily understood?

**Operationalizability:** Can the item be easily translated into a clear survey question?

**Redundancy:** Does the item overlap significantly with others?

3. Decision Outcomes: Following group discussions, experts processed the 86 indicators as follows.

**Retain:** The item was deemed essential, clearly worded, and measurable as a scale item. It was included in the preliminary scale with no or minimal textual changes.

**Modify:** The core concept of the item was essential, but its wording was clarified, broadened, narrowed, or otherwise altered to improve clarity, accuracy, or measurability without changing its fundamental meaning.

**Merge:** Two or more items were combined into a single, more comprehensive item to avoid redundancy and improve parsimony.

**Delete:** The item was considered too specific, not directly measurable via self-report or redundant with other items.

4.Decision Log Table:

| Original Item | Decision | Modified Content / Reason for Deletion | New Item No. |
| --- | --- | --- | --- |
| 1.1.1 Characteristics of the Plateau Environment and Its Effects on the Human Body | Merge | Core knowledge has been integrated to form a comprehensive understanding of the impact on plateau environments. | 4 |
| 1.1.2 Impact of the High-Altitude Environment on Combat Wound Conditions | Merge | As above. | 4 |
| 1.1.3 Impact of the High-Altitude Environment on Medical Rescue Operations | Merge | As above. | 4 |
| 1.1.4 Knowledge Related to Health and Epidemic Prevention in High-Altitude Regions | Modify | Transform specific knowledge points into a self-assessable “Mastery of Knowledge” entry, explicitly listing “Health and Epidemic Prevention” as one of its components. | 5 |
| 1.1.5 High-Altitude Combat Medical Support Knowledge | Retain | Revised Content: Mastering high-altitude medical support nursing knowledge | 5 |
| 1.2.1 Acute Mountain Sickness Prevention, Treatment and Nursing Knowledge | Merge | Combine with 1.2.2 and 1.2.3 into a comprehensive entry titled “Prevention, Treatment, and Care Knowledge for Common Plateau Diseases.” | 6 |
| 1.2.2 High-Altitude Heart Disease Prevention, Treatment and Nursing Knowledge | Merge | As above. | 6 |
| 1.2.3 High-Altitude Polycythemia Prevention, Treatment and Nursing Knowledge | Merge | As above. | 6 |
| 1.3.1 Tactical Combat Casualty Care (TCCC) Theory | Merge | Integrated as a core component of the “Principles and Strategies for Treating Combat Injuries in High-Altitude Environments.” | 7 |
| 1.3.2 Prolonged Casualty Care (PCC) Theory | Merge | As above. | 7 |
| 1.3.3 Preparation and Administration of Common Emergency Medications | Delete | Reason for deletion: This entry places excessive emphasis on specific operational details, whose core principles are already covered in the entries for “Emergency Care” and “Medication Knowledge.” | - |
| 1.3.4 Operation and Maintenance of Common Medical Devices and Equipment | Retain | Revised Content: Master the operation techniques of commonly used clinical equipment and instruments. | 12 |
| 1.3.5 Common Types of Combat Injuries and Their Clinical Manifestations | Modify | 1.3.5 and 1.3.6 have been consolidated into the new entry “Mastering knowledge of battlefield injury care for various regions/primary types of high-altitude environments.” | 8, 9 |
| 1.3.6 Medical Response Knowledge for Injuries Caused by Specific Weapons | Modify | As above. | 8, 9 |
| 1.3.7 Basic Knowledge of Nuclear Radiation Protection | Modify | Revised content: Mastering protection and decontamination techniques for nuclear, biological, and chemical weapons | 17 |
| 1.4.1 Theories of Combat Stress and Their Assessment Methods | Merge | Combine with 1.4.2 and 1.4.3 into “Mastering knowledge of nursing psychology.” | 3 |
| 1.4.2 Theories Related to Post-Traumatic Stress Disorder and Its Assessment Methods | Merge | As above. | 3 |
| 1.4.3 Theories Related to Psychological Intervention | Merge | As above. | 3 |
| 1.4.4 Psychological Effects of High-Altitude Environments and Protective Measures | Merge | Core knowledge has been integrated into the understanding of the overall impact on plateau environments and psychological knowledge. | 3, 4 |
| 2.1.1 Battlefield Environmental Assessment | Modify | Revised content: “Master the content and methods of assessing the condition of casualties on the battlefield.” | 13 |
| 2.1.2 Rapid Casualty Assessment and Examination | Retain | “Master the content and methods of assessing the condition of casualties on the battlefield.” | 13 |
| 2.1.3 Triage Methodology (START) | Retain | “Master the types and techniques of triage in battlefield first aid.” | 14 |
| 2.1.4 Hypoxia Status Evaluation | Merge | As a specific assessment skill, its essence has been incorporated into the “Injury Assessment” entry. | 13 |
| 2.2.1 Assessment of Blood Loss | Merge | As part of its “hemostasis” capability, it is integrated into “identification and management of major hemorrhage” and the higher-level “injury assessment.” | 13 |
| 2.2.2 Identification and Management of Major Bleeding | Merge | Core competencies are retained but incorporated as part of “Basic Combat Casualty Care Techniques,” rather than listed as separate entries. | 15 |
| 2.2.3 Tourniquet Hemostasis (Clamp-Type, Rotating-Type, Standard-Type) | Delete | Reason for deletion: Excessively specific technical procedures. The competency assessment focuses on core competencies rather than detailed technical specifications. This capability is already covered under “Basic Combat Casualty Care Techniques.” | - |
| 2.2.4 Pressure Hemostasis | Delete | As above. | - |
| 2.2.5 Wound Packing | Delete | As above. | - |
| 2.2.6 Pharmacological Hemostasis | Delete | As above. | - |
| 2.2.7 Improvised Hemostasis Methods | Delete | As above. | - |
| 2.3.1 Airway Assessment and Clearance | Merge | Core skills are integrated into “Mastering Emergency Care Procedures.” | 15 |
| 2.3.2 Manual Airway Management Techniques | Delete | Reason for deletion: Excessively specific technical procedures, already covered in the parent entry. | - |
| 2.3.3 Use of a Simple Respirator | Merge | Core skills are integrated into “Mastering Emergency Care Procedures.” | 11 |
| 2.3.4 Mouth-to-mouth resuscitation | Merge | As above. | 11 |
| 2.3.5 Recovery Position/Forward-Leaning Position | Delete | Excessively specific technical procedures are covered in the preceding entry “Mastering Basic Combat Casualty Care Techniques.” | - |
| 2.3.6 Use of the oropharyngeal nasal airway | Delete | Excessively specific technical procedures are covered in the preceding entry “Mastering Basic Combat Casualty Care Techniques—Ventilation.” | - |
| 2.3.7 Tracheal Intubation and Tracheotomy Techniques | Delete | As above. | - |
| 2.3.8 Pneumothorax Identification and Management (Closed, Open, Tension Pneumothorax) | Merge | Recognition capabilities are integrated into “Injury Assessment,” and handling principles are integrated into “Basic Combat Casualty Care Techniques—Ventilation.” | 13, 15 |
| 2.3.9 Oxygen therapy techniques | Merge | Core knowledge and applied skills are integrated into “High-Altitude Medical Support Nursing Knowledge” and “Emergency Nursing Procedures.” | 5, 11 |
| 2.4.1 Timing of CPR Assessment | Merge | All CPR-related skills are consolidated into “Mastery of emergency care techniques (including defibrillation, chest compressions, rescue breathing, Heimlich maneuver, and other common emergency care procedures).” | 11 |
| 2.4.2 Chest Compressions | Merge | As above. | 11 |
| 2.4.3 Manual or Automated Defibrillation | Merge | As above. | 11 |
| 2.4.4 Assessment and Advanced Life Support | Merge | As above. | 11 |
| 2.5.1 Common Bandaging Methods (Triangular Bandages, Bandages, etc.) | Delete | Reason for deletion: Excessively specific technical procedure. This skill is already fully covered under “Mastering basic combat casualty care techniques—bandaging.” | - |
| 2.5.2 Bandaging Techniques for Common Body Areas (Head, Chest, Limbs, etc.) | Delete | As above. | - |
| 2.5.3 Bandaging Techniques for Specialized Areas (Eye, Amputations, etc.) | Delete | As above. | - |
| 2.6.1 Cervical Collar Immobilization | Delete | Reason for deletion: Excessively specific technical procedures. This competency is fully covered under “Mastering Basic Combat Casualty Care Techniques—Immobilization.” | - |
| 2.6.2 Limb Fracture Immobilization | Delete | As above. | - |
| 2.6.3 Spinal Fracture Immobilization | Delete | As above. | - |
| 2.6.4 Pelvic Fracture Immobilization | Delete | As above. | - |
| 2.7.1 Manual Transport Method | Delete | Reason for deletion: Excessively specific technical procedures. This capability is already fully covered under “Mastering basic combat casualty care techniques—transportation.” | - |
| 2.7.2 Stretcher Transport Method | Delete | As above. | - |
| 2.7.3 Special Injury Transport Methods (Spinal, Cranial, etc.) | Delete | As above. | - |
| 2.8.1 Recognition and Management of Shock | Merge | Recognition capabilities are integrated into “Injury Assessment,” while management principles are consolidated into the advanced entries related to “Shock Management.” | 11, 13 |
| 2.8.2 Effective Assessment and Indicators of Shock Resuscitation | Merge | As above. | 11, 13 |
| 2.8.3 Venipuncture and Catheterization | Merge | Integrated as a core skill within “battlefield blood transfusion and infusion techniques.” | 16 |
| 2.8.4 Fluid Resuscitation Strategies and Selection of Resuscitation Fluids | Merge | As above. | 16 |
| 2.8.5 Indications and Contraindications for Intravenous Fluid and Blood Transfusion | Merge | As above. | 16 |
| 2.8.6 Intravenous Infusion Technique in Non-Steady Vibration Dark-Field Environments | Merge | As above. | 16 |
| 2.9.1 Identification and Prevention of Hypothermia | Merge | Has been integrated into the “Injury Assessment.” | 13 |
| 2.9.2 Thermal Protection | Delete | The specific procedures are covered in the basic skills of combat casualty care and fundamental nursing techniques. | - |
| 2.9.3 Passive Warming | Delete | As above. | - |
| 2.9.4 Active Warming | Delete | As above. | - |
| 2.10.1 Nuclear, Chemical, and Biological Weapons First Aid Techniques | Merge | Combined with 2.10.2 and 2.10.3 into “Mastering Protection and Decontamination Techniques for Nuclear, Biological, and Chemical Weapons.” | 17 |
| 2.10.2 Protection against nuclear, chemical, and biological weapons | Merge | As above. | 17 |
| 2.10.3 Decontamination and Isolation of Nuclear, Chemical, and Biological Weapons | Merge | As above. | 17 |
| 2.11.1 Different Evacuation Methods and Their Indications and Contraindications | Retain | - | 18 |
| 2.11.2 Monitoring and Emergency Response During Different Evacuation Methods | Retain | - | 19 |
| 2.11.3 Operation of Life Support Equipment During Medical Evacuation | Retain | - | 20 |
| 2.11.4 Preparation of Air/Ground Transport Documents | Retain | - | 21 |
| 3.1.1 Teamwork and Collaboration Skills | Retain | - | 22, 24 |
| 3.1.2 Mass Casualty Management Capabilities | Modify | Ability is highly correlated with “teamwork” and “emergency decision-making,” and its core elements have been integrated into the relevant entries. | 22, 23 |
| 3.1.3 Effective Communication Skills with Casualties and Team Members | Modify | Integrated into “Comprehension and Expression Skills” and “Interpersonal Skills”. | 25, 26, 27 |
| 3.2.1 Ability to adjust rescue plans in real time based on battlefield conditions | Merge | Combined with 3.2.2 as “Possess the ability to respond and make decisions in emergency situations.” | 23 |
| 3.2.2 Ability to adapt and respond flexibly to sudden and complex issues | Merge | As above. | 23 |
| 3.3.1 Ability to anticipate and identify enemy fire risks | Merge | Combining the content of Sections 3.3.2 and 3.3.3, the core competencies “risk anticipation” and “safety protection” have been integrated into “possessing nursing risk anticipation capabilities,” “ensuring patient safety capabilities,” and “possessing occupational safety protection capabilities.” | 28, 29, 30 |
| 3.3.2 Ability to anticipate and identify potential hazardous environments | Merge | As above. | 28, 29, 30 |
| 3.3.3 Professional safety self-protection capability | Retain | - | 30 |
| 4.1.1 Meeting Military Physical Fitness Test Standards | Modify | Combined with 4.1.2 and 4.1.3 into “Possess good military physical fitness,” “Possess basic wilderness survival knowledge and skills,” and “Possess the ability to perform mental work in high-altitude oxygen-deficient environments.” | 37 |
| 4.1.2 Survival Adaptability | Modify | As above. | 38 |
| 4.1.3 Mental Work Capacity in High-Altitude Hypoxic Environments | Modify | As above. | 39 |
| 4.2.1 Self-Psychological Adjustment Ability | Retain | - | 34 |
| 4.2.2 Coping Ability and Stress Resistance | Retain | - | 35, 36 |
| 4.3.1 National Defense Awareness and Patriotic Consciousness | Retain | - | 31 |
| 4.3.2 Sense of Responsibility and Mission | Modify | Revised content: Possess a strong sense of discipline, responsibility, mission, obedience, and confidentiality. | 32 |
| 4.3.3 Military Knowledge and Military Proficiency | Retain | - | 33 |

The following are additional entries added during the group discussion.

| New entry content | Reason |
| --- | --- |
| 1. Master the fundamental knowledge of nursing | This entry summarizes and integrates the foundational knowledge essential to all technical operations within the Delphi indicators. Expert panel discussions concluded that explicitly evaluating this fundamental dimension is critical to ensuring the comprehensiveness of core competencies. |
| 2. Master specialized nursing knowledge | This entry integrates multiple specialized nursing knowledge domains related to combat casualty care. Experts assert that in high-acuity trauma management, a deep understanding of specialized knowledge forms the foundation for sound clinical decision-making. Therefore, it is essential to evaluate this as a distinct dimension. |
| 10. Master basic nursing procedures | This entry represents the consolidation and refinement of specific operational items under the ‘Technical Expertise’ dimension of the Delphi-derived indicators. To enhance the scale's efficiency, the panel discussion grouped these highly interrelated skills into a single comprehensive entry, whose scope now encompasses multiple specific techniques originally listed under the indicator. |

Ultimately, detailed explanations were provided for every item following the panel discussion to guarantee their clarity, specificity, and operational strength.
