# Supplementary material for: Development and psychometric validation of a core competency scale for military nurses in high-altitude extreme environments
Source: Front Med (Lausanne). 2026 Apr 13;13:1791003. doi: 10.3389/fmed.2026.1791003 (PMC13110977; doi:10.3389/fmed.2026.1791003)
Supplement: Supplementary file 4 [file Table_4.docx]

Core Competency Scale for High-Altitude Extreme Environment Rescue among Military Nurses

| Item No. | Item |
| --- | --- |
|  |  |
| 1 | Mastery of basic nursing professional knowledge (including fundamental nursing theory, vital signs monitoring, aseptic techniques, and other essential knowledge) |
| 2 | Mastery of specialized nursing knowledge (including specialized nursing knowledge in internal/surgical care, critical care, and care for specific diseases) |
| 3 | Mastery of nursing psychology knowledge (including basic knowledge of acute stress reaction, post-traumatic stress disorder, etc.) |
| 4 | Mastery of basic high-altitude-related knowledge (including environmental and climatic characteristics of high-altitude areas and their impact on casualty care) |
| 5 | Mastery of basic high-altitude-related knowledge (including environmental and climatic characteristics of high-altitude areas and their impact on casualty care) |
| 6 | Mastery of prevention, treatment, and nursing knowledge related to common high-altitude diseases (including prevention, early identification, and nursing measures for acute mountain sickness, high-altitude pulmonary edema, and high-altitude cerebral edema) |
| 7 | Mastery of principles and strategies for high-altitude combat casualty care (including Tactical Combat Casualty Care (TCCC) theory, Prolonged Field Care (PFC), and En Route Care (ERC)) |
| 8 | Mastery of nursing knowledge for wounds to various body parts in high-altitude environments (including key nursing points for wounds to the craniocerebral region, maxillofacial area, chest, abdomen, pelvis, limbs, etc.) |
| 9 | Mastery of nursing knowledge for main types of combat wounds in high-altitude environments (including key nursing points for different types of wounds such as blast injuries, impact injuries, frostbite, burns, and biological weapon injuries) |
| 10 | Mastery of basic nursing operational skills (including 27 commonly used clinical basic operational skills such as intravenous infusion, catheterization, sputum suction, wound dressing) |
| 11 | Mastery of emergency nursing operational skills (including commonly used emergency nursing techniques such as defibrillation, cardiac compression, artificial respiration, Heimlich maneuver) |
| 12 | Mastery of operational skills for commonly used clinical equipment and instruments (including operation and maintenance of devices such as ECG monitors, ventilators, defibrillators) |
| 13 | Mastery of the content and methods for assessing casualty conditions on the battlefield (e.g., using the ABCDE method to rapidly identify and manage life-threatening injuries. A (Airway), B (Breathing), C (Circulation), D (Disability/Neurological Function), E (Exposure/Environmental Control), etc.) |
| 14 | Mastery of triage types and techniques for battlefield care (e.g., using the START method to assess and determine the severity of casualties and prioritize evacuation) |
| 15 | Mastery of basic combat casualty care techniques (including the five major techniques: ventilation, hemorrhage control, bandaging, immobilization, and movement/evacuation) |
| 16 | Mastery of battlefield blood transfusion and infusion techniques (including establishing intravenous access under field conditions, standards for blood product transfusion, intraosseous access techniques, and intravenous infusion techniques in non-standard oscillatory dark visual environments) |
| 17 | Mastery of Nuclear, Biological, Chemical (NBC) weapon protection and decontamination techniques (including donning and doffing protective suits, procedures for decontaminating contaminated areas, etc.) |
| 18 | Mastery of different medical evacuation (MEDEVAC) methods and their indications and contraindications (including applicable scenarios and limitations for air and ground transport) |
| 19 | Mastering monitoring and emergency management during different modes of evacuation (including vital sign monitoring, managing barometric pressure changes for pneumothorax patients during air transport, and adjusting analgesic pump dosages during extended ground transport). |
| 20 | Mastery of life-support equipment operation during the MEDEVAC process (including care of various tubes/tubing, hemodynamic monitoring, use of portable ventilators and defibrillators during transport) |
| 21 | Mastery of documentation for air/ground MEDEVAC (including standardized completion and archiving of medical record forms and evacuation handover forms) |
| 22 | Ability for team division of labor and collaboration (refers to the ability to clarify role responsibilities, utilize team spirit, help each other, effectively achieve team goals, and maximize team benefits) |
| 23 | Ability for adaptability and decision-making in emergencies (refers to the ability to quickly analyze problems, develop contingency plans, and execute them in sudden emergency situations) |
| 24 | Ability for team motivation and mobilization (refers to the ability to boost morale, stimulate the team's internal drive, and enhance participation) |
| 25 | Ability for comprehension and expression (refers to the ability to understand the intent of others' expressions and clearly and accurately express one's own intent) |
| 26 | Ability for interpersonal communication (refers to mastering methods and techniques for effective communication with superiors, colleagues, and the sick and wounded) |
| 27 | Ability for non-verbal expression (including the effective use of body language and facial expression management in communication) |
| 28 | Ability to anticipate nursing risks (refers to the ability to identify potential safety hazards in advance, such as falls and aspiration, and take effective nursing measures promptly) |
| 29 | Ability to ensure casualty safety (including preventing secondary injuries to casualties during evacuation and transport) |
| 30 | Ability for occupational safety protection (refers to the ability to ensure one's own safety during treatment, such as correctly wearing protective equipment; strictly adhering to aseptic technique to avoid needlestick injuries; correctly applying mechanical principles when moving patients to reduce personal muscle strain) |
| 31 | Possession of national defense awareness and patriotic consciousness |
| 32 | Possession of strong discipline awareness, sense of responsibility, sense of mission, sense of obedience, and confidentiality awareness |
| 33 | Possession of basic military knowledge and military literacy |
| 34 | Ability for self-psychological adjustment (refers to the ability to promptly adjust and change one's psychological state in the face of sudden situations) |
| 35 | Ability to cope with stress (refers to the ability to adapt to environmental changes and actively respond to pressure and setbacks) |
| 36 | Psychological endurance capacity (refers to the tolerance level for high-intensity stress or stimulation) |
| 37 | Possession of good military physical fitness (refers to achieving passing scores on each military physical fitness assessment) |
| 38 | Possess wilderness survival knowledge and skills (referring to the ability to ensure personal safety and sustain basic survival needs in natural environments) |
| 39 | Possesses the ability to perform mentally demanding tasks in high-altitude hypoxic environments (referring to the maintenance of decision-making, memory, and concentration under low-oxygen conditions) |
