# Supplementary material for: Development and psychometric validation of a core competency scale for military nurses in high-altitude extreme environments
Source: Front Med (Lausanne). 2026 Apr 13;13:1791003. doi: 10.3389/fmed.2026.1791003 (PMC13110977; doi:10.3389/fmed.2026.1791003)
Supplement: Supplementary file 5 [file Table_5.docx]

Additional file 5.Factor loadings of the HA-MNCS (n=88)

| Item | Theoretical knowledge | Professional skill | Comprehensive ability | Personal trait |
| --- | --- | --- | --- | --- |
| 5. Master high-altitude medical care knowledge | 0.861 |  |  |  |
| 4. Master basic knowledge related to high altitudes | 0.835 |  |  |  |
| 3. Master nursing psychology knowledge | 0.819 |  |  |  |
| 6. Master knowledge related to the prevention, treatment, and nursing care of common diseases at high altitudes | 0.813 |  |  |  |
| 9. Master knowledge of nursing care for combat injuries in major types of high-altitude environments | 0.795 |  |  |  |
| 7. Master principles and strategies for treating combat injuries at high altitudes | 0.783 |  |  |  |
| 8. Master knowledge of nursing care for combat injuries in various parts of the body in high-altitude environments | 0.767 |  |  |  |
| 1. Master basic nursing knowledge | 0.685 |  |  |  |
| 2. Master specialized nursing knowledge | 0.666 |  |  |  |
| 12. Master the operation techniques of commonly used clinical equipment and instruments |  | 0.843 |  |  |
| 11. Master emergency nursing operation techniques |  | 0.843 |  |  |
| 10. Master basic nursing operation techniques |  | 0.818 |  |  |
| 13. Master the content and methods of assessing the condition of casualties at the battlefield |  | 0.79 |  |  |
| 15. Master basic battlefield first aid techniques |  | 0.79 |  |  |
| 14. Master the types and techniques of triage classification at the battlefield |  | 0.747 |  |  |
| 19. Master monitoring and emergency response during different evacuation methods |  | 0.745 |  |  |
| 21. Master the writing of wartime nursing documentation |  | 0.742 |  |  |
| 20. Master the operation of life support equipment during medical evacuation |  | 0.73 |  |  |
| 16. Master battlefield blood transfusion and intravenous fluid administration techniques |  | 0.718 |  |  |
| 18. Master the different modes of evacuation and their indications and contraindications |  | 0.634 |  |  |
| 17. Master protection and decontamination techniques for nuclear, biological, and chemical weapons |  | 0.624 |  |  |
| 25. Possess the ability to understand and express oneself |  |  | 0.833 |  |
| 27. Possess the ability to identify potential risks and common safety hazards |  |  | 0.829 |  |
| 26. Possess interpersonal communication skills |  |  | 0.825 |  |
| 22. Possess the ability to divide tasks and collaborate within a team |  |  | 0.786 |  |
| 24. Possess the ability to motivate and mobilize a team |  |  | 0.779 |  |
| 23. Possess the ability to respond and make decisions in emergency situations |  |  | 0.767 |  |
| 28. Possess occupational safety protection capabilities |  |  | 0.75 |  |
| 31. Possess basic military knowledge and military literacy |  |  |  | 0.844 |
| 32. Possess the ability to self-regulate psychological state |  |  |  | 0.838 |
| 30. Possess strong discipline awareness, sense of responsibility, sense of mission, obedience awareness, and confidentiality awareness |  |  |  | 0.819 |
| 34. Possess psychological resilience |  |  |  | 0.816 |
| 33. Possess stress management ability |  |  |  | 0.804 |
| 29. Possess national defense awareness and patriotic consciousness |  |  |  | 0.797 |
| 35. Possess good combat literacy and military physical fitness |  |  |  | 0.777 |
| 36. Possess wilderness survival knowledge and skills |  |  |  | 0.631 |
